# Association of Kaiser sepsis score with confirmed intrauterine infection and inflammation (Triple-I) in clinical chorioamnionitis: a retrospective cohort study

**DOI:** 10.1186/s40748-025-00226-7

**Published:** 2025-10-03

**Authors:** Sophia Rafferty, Amy Heerema-McKenney, Melanie Kasaris, Amanda Smith, Gloria Gordon-Ocejo, Hany Aly, Anirudha Das

**Affiliations:** 1 Cleveland Clinic, Cleveland, OH 44195 USA; 2 Department of Neonatology, Cleveland Clinic Foundation, 9500 Euclid Avenue, Mail Code-M31, Cleveland, OH 44195 USA

## Abstract

**Background:**

Chorioamnionitis is a known independent risk factor for early-onset sepsis (EOS) in infants. In 2015, the term was redefined as “intrauterine inflammation or infection or both” (Triple-I) to improve clinical management of maternal and neonatal infections. This study evaluated the association between the Kaiser sepsis score (KSS), a tool for predicting and managing EOS in newborns, and histopathologic chorioamnionitis (HCA) and confirmed Triple-I.

**Methods:**

This retrospective cohort study included mother-infant dyads with a gestational age of ≥ 34 weeks at birth, delivered between January 2014 and December 2019, with a maternal diagnosis of clinical chorioamnionitis. Receiver operating characteristic (ROC) curves were used to assess the association between the KSS, Triple-I, and HCA.

**Results:**

A total of 230 mother-infant dyads were analyzed, of whom 157 (68.2%) had HCA and 86 (37.3%) had confirmed Triple-I. Infant demographic characteristics were comparable between groups, except for the KSS, which was significantly higher in the Triple-I group [1.22 vs. 0.83, *p* < 0.001; OR 1.21, 95% CI 1.04–1.4]. The KSS demonstrated a strong positive association with confirmed Triple-I (AUC 0.77, 95% CI 0.71–0.83), while its association with HCA was weaker (AUC 0.59, 95% CI 0.51–0.67). At the same KSS threshold, sensitivity for diagnosing Triple-I was higher than for HCA.

**Conclusion:**

KSS showed a stronger association with confirmed Triple-I compared to HCA in mothers with clinical chorioamnionitis, suggesting that Triple-I is a better predictor of EOS risk.

## Introduction

Chorioamnionitis refers to infection or inflammation of the placenta and/or the amniotic fluid [1]which complicates 1–4% of all births in the United States [2]. Often Clinical chorioamnionitis and the term Chorioamnionitis is used interchangeably and is an independent risk factor for intubation of the infant in the delivery room, pneumonia, neonatal seizures, and Neonatal Early Onset Sepsis (EOS) [3–5]. Neonatal EOS is relatively rare but a serious complication in newborn infants [6]. The Kaiser sepsis score (KSS), also known as Kaiser Permanente Neonatal Early-Onset Sepsis (EOS) Calculator is a tool to estimate the risk of EOS in newborns [7, 8]. The KSS is used to determine the risk for infection in neonates and was found to correlate with Histopathologic Chorioamnionitis (HCA), including Funisitis, which is frequently considered when determining the need and duration of empirical antibiotic therapy in neonates without culture-positive sepsis [9]. It is essential to mention that the information on the diagnosis of HCA is not available immediately at birth as it takes time to complete the pathological examination of the placenta. Providers frequently consider the presence or absence of HCA, including Funisitis, to determine the need and duration of empirical antibiotic use in neonates without culture-positive sepsis [10].

A new term, “Intrauterine inflammation or infection or both (Triple-I),” was introduced in a workshop by experts from the National Institute of Child Health and Human Development (NICHD) [11]. This new definition, Triple-I, is considered more specific than previous terminology of clinical chorioamnionitis. Since both HCA and Triple-I are associated with increased chances of EOS in newborns and the KSS is the basis of the prophylactic management of EOS in infants, we sought to determine the association of KSS at birth with both HCA and Triple-I.

## Methods

### Study design

This was an observational retrospective cohort study. The Institutional Review Board (IRB) approved this observational retrospective cohort study.

### Settings

The study was conducted in two level three Neonatal Intensive Care units in an urban setting. All mothers with clinical chorioamnionitis who met the inclusion criteria were identified, and data were extracted from the electronic medical records of those who gave birth between January 2014 and December 2019.

Participants: Mother-infant dyads fulfilling the following study criteria were included.

#### Inclusion criteria

- Infants born at 34 weeks 0 days or more.
- All infant-mother dyads with a peripartum maternal diagnosis of clinical chorioamnionitis.

#### Exclusion criteria

- Dyads without sufficient documentation of criteria for determining the KSS or making a diagnosis of Triple-I.

### Variables

Demographic variables of the mother and the infant, as well as variables required to determine the infant’s KSS at birth and a diagnosis of Triple-I, were included.

### Definitions

*Clinical chorioamnionitis* is defined by the presence of fever, uterine fundal tenderness, maternal tachycardia (> 100/min), fetal tachycardia (> 160/min), and purulent or foul amniotic fluid [12]. A diagnosis of Clinical chorioamnionitis does not correspond to HCA or confirmed Triple-I but this cohort has increased chances of being diagnosed with HCA or positive Triple-I which is why we considered it as an inclusion criteria.

*Histopathologic Chorioamnionitis (HCA*): It is defined by pathologists as an intrauterine inflammatory condition characterized by acute granulocyte infiltration into the fetal–maternal or into the fetal tissues [13].

Early Onset Sepsis of the neonate (EOS): This is defined as neonatal sepsis occurring in the first three days of life (although some may include the first 7 days of life) [14].

*Kaiser sepsis score (KSS)*: The score can be calculated online (https://neonatalsepsiscalculator.kaiserpermanente.org/InfectionProbabilityCalculator.aspx) and by submitting objective maternal risk factors and birth data: gestational age, highest maternal intrapartum temperature, length of rupture of membranes, GBS status, and use of intrapartum antibiotics [7, 8]. The score was calculated solely based on these criteria without considering the clinical evaluation part.

*Intrauterine inflammation or infection or both (Triple-I)*:

*Suspected Triple-I* is diagnosed when there is a maternal fever without an apparent source along with any one of the following criteria: (1) Baseline fetal tachycardia, defined as a fetal heart rate greater than 160 beats per minute sustained for 10 min or longer (excluding accelerations, decelerations, and periods of marked variability). (2) A maternal white blood cell count greater than 15,000 cells per mm³ in the absence of corticosteroid administration. (3) The presence of definite purulent fluid from the cervical os.

*Confirmed Triple-I* requires all the criteria for suspected Triple I plus objective laboratory evidence of infection. This may include: (1) A positive amniotic fluid Gram stain for bacteria, low amniotic fluid glucose (e.g., ≤ 14 mg/dL), a high white cell count in amniotic fluid (e.g., > 30 cells/mm³ in the absence of a bloody tap), or a positive amniotic fluid culture result or *(2)* Histopathological evidence of infection, inflammation, or both in the placenta, fetal membranes, or umbilical cord vessels (Funisitis).

In this study, a diagnosis of Confirmed Triple-I is equivalent to Triple-I positive.

### Ethical considerations

The Institutional Review Board (IRB) approved this observational retrospective cohort study. The need for consent was waived.

### Data source/measurements

Data were obtained from a retrospective review of infant charts included in the study. The variables’ measurements were standardized and self-explanatory. EOS risk was calculated using the Neonatal Early-Onset Sepsis Calculator (KSS) [15] online tool. The EOS risk at birth (baseline risk) was considered 0.5/1000 live births (Centers for Disease Control and Prevention—CDC National Incidence) for all calculations.

### Statistical methods

SPSS software was used for data analysis (IBM SPSS version 27, 2020). We used the convenience sampling method. Median and interquartile ranges were reported to describe the demographic and outcome variables. The Receiver Operating Characteristic (ROC) curve was utilized to determine the strength of association between KSS for both diagnosis of Chorioamnionitis (Triple-I or HCA). Median, interquartile ranges, and percentages were reported as applicable to describe the demographic and outcome variables. Missing data were not analyzed.

### Sample size estimate

To obtain an Area Under the Curve (AUC) of 80%, where an estimated 40% of the sample is positive for Triple-I, with a confidence interval width of 0.12 and a confidence level of 0.95, a sample size of 239 is needed [16]. We initially collected 241 samples but excluded 11 subjects as they did not meet the criteria.

## Results

The rate of clinical chorioamnionitis was 3.6% in the population during the study period. A total of 230 mother-infant dyads met the inclusion criteria, out of which 157 (68.2%) had histopathologic chorioamnionitis (HCA), while 86 cases (37.3%) were positive for Triple-I. Out of 157 cases of HCA, 77 (49%) were also positive for Triple-I. The demographic comparison of dyads with HCA positive and Triple-I positive is shown in Table 1.

**Table 1:** Demographic characteristics of the cohort

	HCA (*n* = 157) *	Triple-I (*n* = 86) *	*P* value
Birth weight (Kg)	3.38 (3.12–3.72)	3.39 (3.14–3.76)	0.78
Gestational Age (weeks)	39 (39–40)	39 (39–40)	0.56
Male (%)	42	44.2	0.78
Maternal Age (years)	28 (24–32)	29 (25–34)	0.36
Gravida (%)	1 (1–2)	1 (1–2)	
Days of antibiotics (days)	2 (1–2)	2 (1-2.25)	0.54
Positive CRP (%)	28.7	29.1	0.97
Phototherapy (%)	7.6	10.5	0.73
Sepsis workup (%)	79	76.7	0.74
NICU admission (%)	65	70.9	0.39
Length of stay (days)	3 (3–5)	3 (2-4.25)	0.82
Kaiser sepsis score**	0.83 (0.34–1.52)	1.22 (0.74–2.37)	< 0.001

*Median and interquartile range or %, as applicable. **P < 0.001 (OR 1.21, 95% CI 1.04 to 1.4).

There was no significant difference in the demographic characteristics of the infants between the two groups (HCA vs. Triple-I) except the KSS. However, the rate of positive CRP, phototherapy, and NICU admission were higher in the Triple-I group, but the difference was not significant. The KSS at birth was significantly higher in the Triple-I group [1.22 vs. 0.83, *p* < 0.001 (OR 1.21, 95% CI 1.04 to 1.4)] compared to the HCA group. There were no significant differences between the amniotic fluid culture positivity rate between the HCA positive and Triple-I groups.

**Fig. 1:**
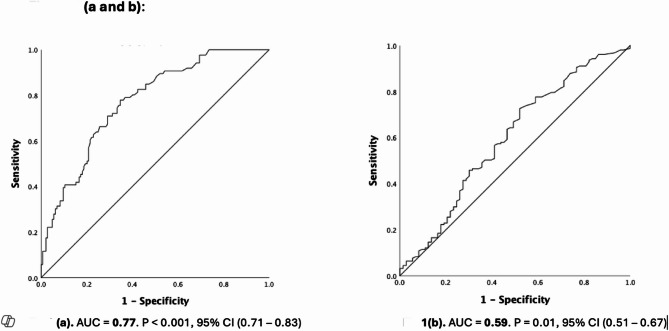
ROC curve showing the prediction of confirmed Triple-I vs. Histopathologic Chorioamnionitis (HCA) using KSS EOS risk estimation

The area under the curve (AUC) of the KSS for Triple-I was 0.77 [95% Confidence Interval (CI) 0.71–0.83], showing a strong positive association, while the AUC of the KSS for HCA was 0.59 (95% CI 0.51–0.67), which is significant but weaker (Fig. 1). At a KSS of 1, sensitivity to correctly diagnose HCA was 43.3% and specificity of 69.9% with a Youden’s index of 0.13. At the same KSS of 1, the sensitivity to correctly diagnose Triple-I was 64% and a specificity of 75%, with a higher Youden’s index of 0.39. Thus, the KSS had a higher sensitivity and specificity in diagnosing Triple-I than HCA.

## Discussion

Our study demonstrated that the KSS is more strongly associated with confirmed Triple-I than HCA. Therefore, the KSS for late preterm and term infants at birth may help obstetric providers decide whether to send the placenta for evaluation and help neonatal providers determine the use and length of antibiotic therapy in suspected and culture-negative EOS.

Not all cases of a diagnosis of clinical chorioamnionitis in the mother are associated with HCA. HCA was present in 84% of cases of maternal clinical chorioamnionitis in a study of 119 infants by Ji (2019) and 82.1% in another study [17, 9]. In our study, the rate of HCA in clinical chorioamnionitis was 68.2%, slightly lower than in the previous studies. The difference could result from including preterm infants < 34 weeks in the other two studies, where the incidence of HCA is higher.

A study by Coleman (2019) showed the rate of Triple-I as 35.5% in mothers diagnosed with clinical chorioamnionitis at a gestation of 35 weeks or more [18]. Another study showed a Triple-I rate of 28% but included gestational ages of 37 weeks or more. In this study, which included infants delivered at 34 weeks or more, 37.3% of the subjects with clinical chorioamnionitis were diagnosed as confirmed triple-I, which is consistent with the findings of the previous studies.

Coleman (2019) demonstrated a lack of correlation between the KSS and Triple-I in their study [18]. One major drawback of their study was that the sample was entirely drawn from the NICU admissions, which is a source of bias as those infants admitted may have had a diagnosis other than sepsis. This contrasts with our study, where the denominator was all newborns with a maternal diagnosis of clinical sepsis, with 65% of the infants eventually being admitted to the NICU. This difference in methodology is vital because Puopolo (2011), in their study estimating the probability of neonatal EOS, considered all newborn infants regardless of NICU admission, similar to our study [7]. Therefore, this study’s outcome is accurate and more generalizable to all newborns.

Since the incidence of blood culture-positive neonatal EOS has reduced drastically, there is a lack of consensus on how to treat culture-negative sepsis [19]. Infants with abnormal laboratory results suggestive of infection or maternal factors such as clinical and histopathologic chorioamnionitis, including funisitis, are utilized to determine the need. Considering Triple-I, rather than clinical chorioamnionitis or HCA, to assess the management of suspected EOS, as shown in this study, may help reduce excess antibiotic utilization.

While the incidence of culture-positive sepsis is low, most antibiotics (6–16 times) are currently used for culture-negative sepsis [10]. The decision to treat the infant for more than 36–48 h is based on several clinical and biochemical factors for which there are currently no consensus guidelines. Some providers do consider maternal chorioamnionitis (with or without Funisitis) as a guide to making that decision. This study demonstrates that Triple-I may be better than HCA in those circumstances.

Infants with positive maternal Triple-I had a higher rate of NICU admissions and positive CRP but a lower rate of sepsis workup. This questions the criteria for performing a sepsis workup in newborns, suggesting that multiple other variables may be associated with sepsis evaluation in newborns.

### Limitations

Retrospective studies have inherent biases which are challenging to identify and eliminate. Although we considered the KSS to be 0.5/1000 live births per CDC, the incidence of culture-positive EOS could be higher in this population, which we did not consider.

## Conclusion

KSS showed a stronger association with confirmed Triple-I compared to HCA in mothers with clinical chorioamnionitis, indicating that Triple-I may be a stronger predictor of EOS in newborn infants. Further studies are needed to validate the strength of this association.

## Data Availability

The datasets generated and/or analyzed during the current study are not publicly available due to Cleveland Clinic Foundation policies but are available from the corresponding author upon reasonable request.
